# An updated atlas of human helminth infections: the example of East Africa

**DOI:** 10.1186/1476-072X-8-42

**Published:** 2009-07-09

**Authors:** Simon Brooker, Narcis B Kabatereine, Jennifer L Smith, Denise Mupfasoni, Mariam T Mwanje, Onésime Ndayishimiye, Nicholas JS Lwambo, Deborah Mbotha, Peris Karanja, Charles Mwandawiro, Eric Muchiri, Archie CA Clements, Donald AP Bundy, Robert W Snow

**Affiliations:** 1 Department of Infectious and Tropical Diseases, London School of Hygiene and Tropical Medicine, UK; 2 Kenya Medical Research Institute (KEMRI)-Wellcome Trust Research Programme, Nairobi, Kenya; 3 Vector Control Division, Uganda Ministry of Health, Kampala, Uganda; 4 Neglected Tropical Disease Control Programme, Access Project, Kigali, Rwanda; 5 Division of Vector Borne Diseases, Kenya Ministry of Health, Nairobi, Kenya; 6 Projet Maladies Tropicales Négligées, Bujumbura, Burundi; 7 National Institute for Medical Research, Mwanza, United Republic of Tanzania; 8 Eastern and Southern Africa Centre of International Parasite Control, KEMRI, Nairobi, Kenya; 9 School of Population Health, University of Queensland, Australia; 10 Human Development Network, The World Bank, Washington DC, USA; 11 Centre for Tropical Medicine, University of Oxford, UK

## Abstract

**Background:**

Reliable and updated maps of helminth (worm) infection distributions are essential to target control strategies to those populations in greatest need. Although many surveys have been conducted in endemic countries, the data are rarely available in a form that is accessible to policy makers and the managers of public health programmes. This is especially true in sub-Saharan Africa, where empirical data are seldom in the public domain. In an attempt to address the paucity of geographical information on helminth risk, this article describes the development of an updated global atlas of human helminth infection, showing the example of East Africa.

**Methods:**

Empirical, cross-sectional estimates of infection prevalence conducted since 1980 were identified using electronic and manual search strategies of published and unpublished sources. A number of inclusion criteria were imposed for identified information, which was extracted into a standardized database. Details of survey population, diagnostic methods, sample size and numbers infected with schistosomes and soil-transmitted helminths were recorded. A unique identifier linked each record to an electronic copy of the source document, in portable document format. An attempt was made to identify the geographical location of each record using standardized geolocation procedures and the assembled data were incorporated into a geographical information system.

**Results:**

At the time of writing, over 2,748 prevalence surveys were identified through multiple search strategies. Of these, 2,612 were able to be geolocated and mapped. More than half (58%) of included surveys were from grey literature or unpublished sources, underlining the importance of reviewing in-country sources. 66% of all surveys were conducted since 2000. Comprehensive, countrywide data are available for Burundi, Rwanda and Uganda. In contrast, information for Kenya and Tanzania is typically clustered in specific regions of the country, with few records from areas with very low population density and/or environmental conditions which are unfavourable for helminth transmission. Information is presented on the prevalence and geographical distribution for the major helminth species.

**Conclusion:**

For all five countries, the information assembled in the current atlas provides the most reliable, up-to-date and comprehensive source of data on the distribution of common helminth infections to guide the rational implementation of control efforts.

## Background

Helminth infections are parasitic worms found in the intestinal tract, urinary tract or blood of humans. The helminth species that cause the greatest human morbidity are the schistosomes, intestinal nematodes (or commonly called soil-transmitted helminths, STH), and tissue nematodes, including human filariae that cause lymphatic filariasis and onchocerciasis [1]. Although helminth infections can infect all members of a population, it is clear that there are specific groups who are at greater risk of morbidity than others, and who are more vulnerable to the harmful effects of chronic infections [2,3]. For schistosomes and STH, the most vulnerable groups are school-aged children and women of child-bearing age, including adolescent girls. Fortunately, much of the morbidity associated with infection can be reversed with the use of effective anthelmintic drug treatments [4,5]. The World Health Organization (WHO) recommends mass drug administration with praziquantel (for schistosomes) and albendazole or mebendazole (for STH) wherever the prevalence of infection exceeds 10%, and has the target of deworming at least 75% of school-aged children and other high-risk groups by 2010 [6]. This goal has encouraged many countries to establish national action plans and programmes for controlling schistosomes and STH. However, the implementation of such programmes requires reliable and up-to-date information on the geographical distribution of infection in order to (i) to guide control to areas in greatest need and (ii) estimate drug requirements.

Previous efforts to develop maps of helminth distributions have included a 1987 global atlas of schistosomiasis [7] and older regional atlases of health and disease, for example, in East Africa [8,9]. Since the mid 1990s, there has been a renaissance in disease mapping, particularly through the use of geographic information systems (GIS) which have made data integration and mapping more accessible and reliable. A principal advantage of a GIS platform is that it facilitates regular updating of information and provides a ready basis for analysis and statistical modelling of spatial distributions, with recent GIS applications focusing on animal diseases [10-12], tick-borne diseases [13], human African trypanosomiasis [14], rabies [15] and malaria [16,17]. In 1999, an international initiative was launched to collate available survey data on schistosomes and STH into a single GIS platform [18]. An important early observation of the work was the paucity of empirical data for large areas of Africa: by 2000 survey data were available for only a third of all districts [18]. In recent years, however, there has been an increase in political, financial and technical support for helminth control, including support for helminth prevalence surveys. East Africa in particular has benefitted from such support, with national programmes launched in Uganda (2003); Tanzania (2003); Burundi (2006); Rwanda (2007); and Kenya (2009). National programmes have been established in the first four countries with support from the Schistosomiasis Control Initiative [19,20] and the Global Network for Neglected Tropical Diseases [21], and in Kenya, with support from the national government, through World Bank and Department for International Development funding, and from Deworm the World [22]. The main strategy of all these national programmes is the delivery of deworming through the school system, which has been demonstrated to reduce infection and morbidity cost-effectively [23-25] and enhance educational outcomes [26] in the region. Generally, in schools where prevalence is 10–50% mass treatment of all school children is conducted every other year, and in schools where prevalence exceeds 50%, annual deworming is conducted at least once a year.

The cost-effective design of all these programmes is dependent upon the availability of accurate and current information on the geographical distribution of infection. This paper reports on recent progress made on updating of an atlas of human helminth infection [18,27,28]. The atlas is initially focusedon the most common helminths of humans which are both highly prevalent and the cause of the greatest burden, namely schistosomiasis and STH [1]. The methods and approaches used to develop the database are detailed, as are the GIS approaches used to map the distribution of the major schistosomes and STH. We then provide new data on the East African countries of Burundi, Kenya, Rwanda, Tanzania and Uganda, and discuss the implications of our findings for ongoing helminth mapping and control.

## Methods

### Mapping helminth transmission

The burden of helminth infection in a given community can be measured by two indicators, either the intensity of infection or the prevalence of infection. Intensity of infection is a measure of the number of eggs per gram of faeces (for STH and *Schistosoma mansoni*) or eggs per 10 ml of urine (for *S. haematobium*), and is a key determinant of transmission dynamics within communities and the risk of morbidity among individuals [29]. Measuring intensity requires time-consuming, quantitative laboratory methods and consequently is not routinely assessed in field surveys. The more easily collected indicator is the prevalence of infection: the proportion of sampled individuals who have one or more eggs detected in their stool or urine sample. In light of the relative simplicity of measuring prevalence, WHO recommends its use to determine the need for control, with mass treatment of whole populations recommended where prevalence exceeds 10% [6].

### Data searches

Relevant information on the prevalence of each of the major schistsome and STH species was identified through a combination of (i) searches of electronic bibliographic databases, (ii) manual searches of local archives and libraries, and (iii) direct contact with researchers. An initial systematic search of published articles was undertaken in 1999 and repeated periodically between 2007 and 2009. The online databases PubMed (1980–2009), MEDLINE (1980–2009) and EMBASE (1980–2009) were used to identify relevant studies for STH, using the Medical Subject Headings (MSHs) hookworm, ascarisiasis, trichuriasis, *Necator americanus, Ancylostoma duodenale*, *Ascaris lumbricoides, Trichuris trichiura*, intestinal parasites, geohelminths, or soil-transmitted helminths AND Burundi, Kenya, Rwanda, Tanzania or Uganda. For relevant studies on schistosomiasis, the terms schistosomiasis, bilharzia, *Schistosoma mansoni*, and *Schistosoma haematobium *were used. All permutations of MSHs were entered and each search was conducted twice to ensure accuracy. The search included non-English language papers. The abstracts of returned articles were then reviewed, and if they did not explicitly report prevalence surveys, they were discarded. All articles were retrieved where the abstract indicated that they contained potentially useful information. We also reviewed reference lists of identified articles and key reviews. Where suitable papers did not provide information in a relevant format, authors were contacted by e-mail and requested to provide relevant data summaries. The second search strategy involved the identification of 'grey' literature sources, including university theses, unpublished surveys and Ministry of Health (MoH) archives. For Kenya, the archives of the Division of Vector Borne Diseases (DVBD) of the Ministry of Health provided a particularly important source of information. The third source of information included personal contact with researchers known to have undertaken surveys in East Africa.

### Geo-positioning procedures

The processes used to determine the longitude and latitude of surveyed schools and communities – termed geo-positioning – are detailed by Guerra et al. [30], who also outlined the challenges involved. In brief, a variety of approaches and sources of information were employed, with use of Microsoft Encarta Premium Edition 2007 as the gold standard for geo-positioning. Other electronic sources of information included GeoNet Names Server [31], Alexandria Digital Library [32], Google Earth [33], Wikipedia [34], and Maplandia [35]. Locations identified from one source were subsequently cross-checked against other sources. Ideally, surveys were located to a point location. However, in certain instances surveys were located to a wide-area polygon (10–25 km^2 ^area), where the centroid of the area or polygon was used. In addition, the sub-national first and second administrative unit was derived for each survey location using the United Nation's Second Administrative Boundaries (SALB) database [36]. This database was used to provide a standard, as sub-national boundaries are constantly changing.

### Data selection and entry

Pre-determined inclusion criteria were applied to information identified through searches. First, only cross-sectional prevalence surveys were included in the database. Multiple surveys may be available from the same location but surveyed at different times; these surveys were included as separate entries. Data were excluded if based on hospital or clinic surveys, post-intervention surveys, or surveys among sub-populations such as among refugee, prison or nomadic populations. Survey data were also excluded if only prevalence was reported without provision of the denominator, or if there were errors in the calculations presented. Finally, studies that could not be geo-positioned to actual location or to a wide-area polygon were excluded.

Each source of information was reviewed and the data extracted into a standardized Microsoft Excel database. Abstracted data included details on the source of the data, location of survey, characteristics of the surveyed population, survey methodology and the number of individuals examined and the number positive for each helminth species. Due to the coprological (diagnostic) method typically used in field surveys, the two species of hookworm (*N. americanus *and *A. duodenale*) could not be distinguished. The few coprological surveys in East Africa which have undertaken differential diagnosis indicate that both species can occur, but that there is a predominance of *N. americanus *in the region [37-39].

### Analysis and mapping

The characteristics of included surveys were summarized by country according to survey population and survey methods. For each helminth species, the median estimate of infection prevalence along with the inter-quartile range, minimum and maximum were calculated according to first-level administrative boundaries for Kenya, Tanzania and Uganda, where detailed sub-national data exist. The decision whether a survey was located to a rural, peri-urban or urban area was derived from the Global Rural Urban Mapping Project (GRUMP) urban extent mask [40]. Population density was derived from a 100 m gridded population map produced using population census data and landcover data [41]. Geo-positioned surveys were imported into Arc Map 9.3 (ERSI, Redlands, CA, USA) which was used to generate the prevalence maps. Point estimates of prevalence were categorized according to WHO prevalence thresholds used to denote treatment requirements [6], with an added category denoting zero prevalence: 0, 0.1–9.9, 10–49.9 and 50–100%.

## Results

The combined search strategies identified 2,748 survey locations for East Africa that were eligible for inclusion. Of these surveys, 2,612 (95.1%) were geographically positioned to an actual longitude and latitude and are included in the current atlas. This includes 41 surveys undertaken in Burundi, 1,329 in Kenya, 138 in Rwanda, 410 in Tanzania, and 694 in Uganda, conducted between 1980 and 2009, representing the examination of 360,276 individuals. Summary characteristics of included surveys are reported by country in Table 1. Overall, data extracted from published papers accounted for 1,096 (42.0%) of all data points, and was the main source of data for Burundi (100%), Tanzania (91.5%) and Uganda (57.5%). In Rwanda, unpublished sources were of greatest importance (Mupfasoni et al. unpublished). In Kenya, the MoH was an important source of prevalence data for both STH and schistosomiasis, with 68.0% of included surveys conducted by the Division of Vector Borne Diseases (DVBD). The MoH was also an important source of data for Uganda, although most of these data were also published [42,43]. Personal communication with authors was a valuable source of survey data, accounting for 544 (20.8%) of surveys results overall. Of the included geo-located surveys, the median sample size was 66, with the range 20 to 4751. The majority (90.4%) of surveys was conducted in schools and is representative of school-age children. Both community-based and school-based surveys were identified for all countries.

**Table 1:** Description of prevalence surveys included in the Atlas

	Burundi	Kenya	Rwanda	Tanzania	Uganda	Total
Total number of surveys identified	41	1,329	138	410	694	2,612
Number individuals surveyed	21,971	212,910	20,665	49,638	55,092	360,276
Number of surveys reporting data on STH	31 (*75.6*)	856 (*64.4*)	134 (*97.1)*	321 (*78.3*)	606 (*87.3*)	1,948 (*74.6*)
Hookworm	31 (*75.6*)	847 (*63.7*)	134 (*97.1*)	321 (*78.3*)	571 (*82.3*))	1,904 (*72.9*)
*Ascaris lumbricoides*	31 (*75.6*)	842 *(63.4*)	134 (*97.1*)	319 (*77.8*)	572 (*82.4*)	1,898 (*72.7)*
*Trichuris trichiura*	31 (*75.6*)	785 (*59.1*)	134 (*97.1*)	319 (*77.8*)	571 (*82.3*)	1,840 (*70.4*)
Number of surveys reporting data on schistosomiasis	41 (*100*)	1,175 (*88.4*)	138 (*100*)	357 (*87.1*)	694 (*100*)	2,405 (*92.1*)
*Schistosoma haematobium*	31(75.6)	625 (*47.0*)	134 (*97.1*)	345 (*84.2)*	66 (9.5)	1,170 (*44.8*)
*S. mansoni*	41 (*100*)	661 (*49.7*)	138 (*100*)	267 (*65.1*)	656 (*94.5*)	1,763 (*67.5*)
						
Sources of survey						
Published paper	41 (*100*)	277 (*20.8*)	4 (*2.9*)	375 (*91.5*)	399 (*57.5*)	1,096 (*42.0*)
Unpublished report	0 (*0.0*)	39 (*2.9*)	0 (*0.0*)	3 (*0.7*)	0 (*0.0*)	42 (*1.6*)
MoH report	0 (*0.0*)	904 (*68.0*)	0 (*0.0*)	0 (*0.0*)	0 (*0.0*)	904 (*34.6*)
Personal communication	0 (*0.0*)	108 (*8.1*)	134 (*97.1*)	7 (*1.7*)	295 (*42.5*)	544 (*20.8*)
Thesis	0 (*0.0*)	1 (*0.1*)	0 (*0.0*)	25 (*6.1*)	0 (*0.0*)	26 (*1.0*)
						
Stool examination method						
Kato-Katz	41(*100*)	471 (*51.3*)	134 (*97.1*)	328 (*100*)	652 (*99.4*)	1,626 (*78.1*)
Formalin-ether and other concentration techniques^1^	0 (*0.0*)	22 (*2.4*)	0 (*0.0*)	0 (*0.0*)	1 (*0.2*)	2 (*1.1*)
Direct smear	0 (*0.0*)	26 (*2.8*)	0 (*0.0*)	0 (*0.0*)	0 (*0.0*)	26 (*1.3*)
Unknown	0 (*0.0*)	401 (*43.6*)	4 (*2.9*)	0 (*0.0*)	3 (*0.5*)	408 (19.6)
						
Urine examination method						
Urine filtration	31(*100*)	136 (*21.7*)	134 (*100*)	316 (*91.6*)	38 (*57.6*)	624 (*53.3*)
Centrifugation and sedimentation	0 (*0.0*)	0 (*0.0*)	0 (*0.0*)	2 (0.6)	0 (*0.0*)	2 (*0.2*)
Reagent strips	0 (*0.0*)	47 (*7.5*)	0 (*0.0*)	26 (*7.5*)	0 (*0.0*)	73 (*6.2*)
Circulating cathodic antigens	0 (*0.0*)	0 (*0.0*)	0 (*0.0*)	1 (*0.3*)	27 (*40.9*)	28 (*2.4*)
Unknown	0 (*0.0*)	442 (*70.7*)	0 (*0.0*)	0 (*0.0*)	1 (*1.5*)	443 (*37.9*)
						
Location of surveys						
Schools	32 (*78.1*)	1,224 (*92.1*)	134 (*97.1*)	383 (*93.4*)	589 (*84.9*)	2,362 (*90.4*)
Communities	0 (*0.0*)	78 (*5.9*)	4 (*2.9*)	27 (*6.6*)	100 (*14.4*)	209 (*8.0*)
Other	9 (*22.0*)	27 (*2.0*)	0 (*0.0*)	0 (*0.0*)	5 (*0.7*)	41 (*1.6*)
Rural	32 (*78.1*)	1,251 (*94.1*)	130 (*94.2*)	373 (*91.0*)	631 (*90.9*)	2,417 (*92.5*)
Urban	5 (*12.2*)	25 (*1.9*)	6 (*4.4*)	21 (*5.1*)	55 (*7.9*)	112 (*4.3*)
Peri-Urban	4 (*9.8*)	53 (*4.0*)	2 (*1.5*)	16 (*3.9*)	8 (*1.2*)	83 (*3.2*)
						
Age ranges examined						
0–4 years	0 (*0.0*)	37 (*2.8*)	0 (*0.0*)	23 (*5.6*)	24 (*3.5*)	84 (*3.2*)
5–16 years	41 (*100*)	1,209 (*91.0*)	134 (*97.1*)	366 (*89.3*)	593 (*85.5*)	2,343 (*89.7*)
16+ years	0 (*0.0*)	3 (*0.2*)	0 (*0.0*)	6 (*1.5*)	0 (*0.0*)	9 (0.3)
All ages	0 (*0.0*)	80 (*6.0*)	4 (*2.9*)	15 (*3.7*)	77 (*11.1*)	176 (*6.7*)

**Figure 1:**
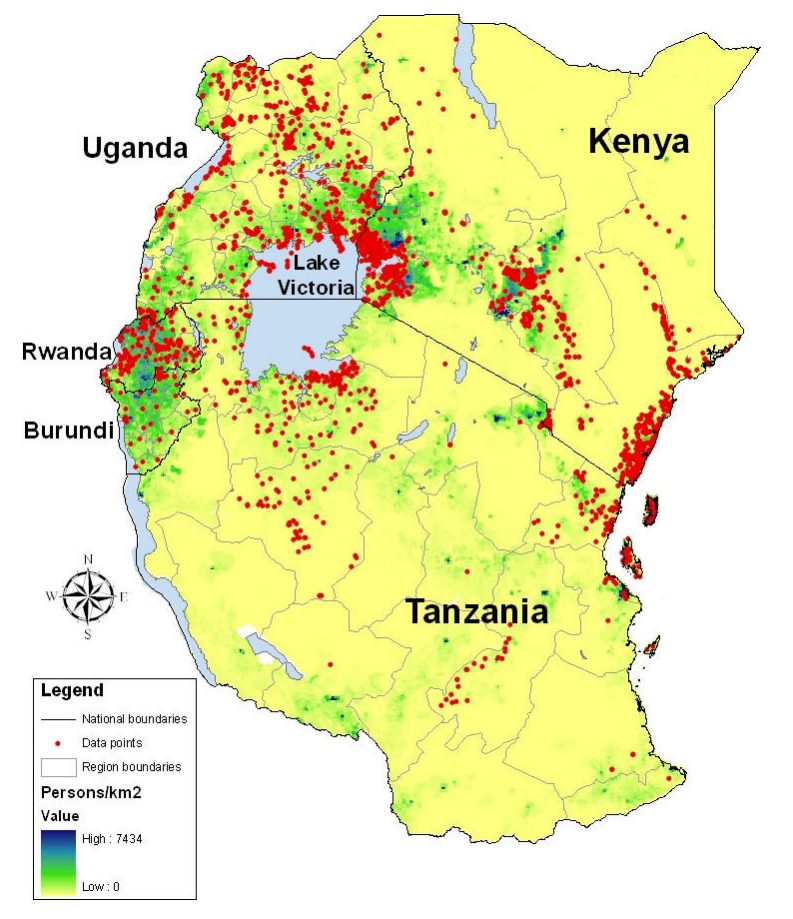
**The spatial distribution of survey sites included in the East African Atlas**. The Atlas currently includes 2,612 surveys conducted between 1980 and 2009. First-level administrative boundaries are indicated in grey. Population density is based on a 100 m gridded population surface [41].

**Figure 2:**
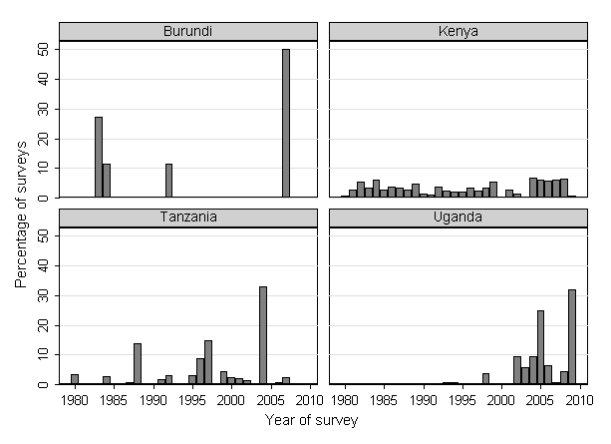
**Time period of included surveys**. The number of prevalence surveys identified by year in Burundi, Kenya, Tanzania and Uganda, East Africa, 1980–2009. The majority (134/138) of surveys in Rwanda were conducted in 2008 and therefore are not presented here. The graphs show a recent increase in the number of surveys conducted since 2000 in East Africa, especially in Tanzania and Uganda.

Figure 1 shows how the geographical distribution of records varies amongst countries. Burundi, Rwanda and Uganda have data from all regions. In Kenya, records were clustered along the coast and Tana River, and in the centre and west of the country; few records were available for northeastern or southern Kenya. In Tanzania, records were mainly from northeast Tanzania or from around Lake Victoria. Although it cannot be assumed that the distribution of these records reflects where helminth infections are a problem, many areas where no records exist have very low population densities (Figure 1). Figure 2 describes the number of surveys conducted by year and by country since 1980. Substantial numbers of surveys have been conducted annually in Kenya throughout this time period. In Burundi very few surveys have been conducted, and in Uganda and Tanzania most of the surveys have been conducted since 2000.

Of the included records, 80.4% provided information on the stool examination method employed and 62.1% on urine method employed. Failure to report the method employed was a particular issue among MoH-DVBD surveys in Kenya, with 74.0% of surveys not stating a stool examination method and 95.3% not stating a urine examination method. Where stated, the most common methods were Kato-Katz (97.1%) and urine filtration (85.8%) method for stool and urine, respectively.

**Figure 3:**
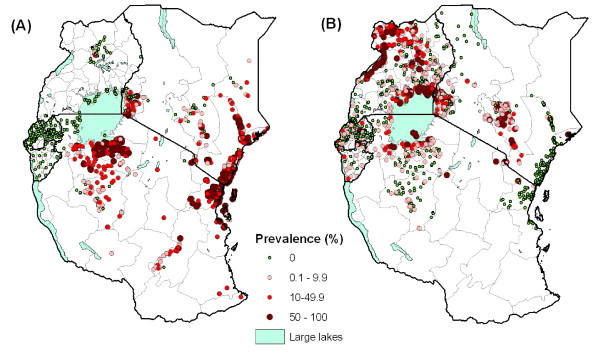
**The known geographical distribution of schistosomiasis in East Africa**. The geographical distribution of (A) *Schistosoma haematobium *and (B) *S. mansoni *infection, based on available survey collected between 1980 and 2009, and categorized according to WHO prevalence thresholds (*n *= 2,405). First-level administrative boundaries are indicated in grey. *S. mansoni *infection is most prevalent around Lake Victoria basin, North-west Uganda and the central highlands of Kenya. In contrast, *S. haematobium *infection is distributed along the Kenyan and Tanzanian coast, Tana River in Kenya and Lake Victoria in Kenya and Tanzania, but absent from Uganda.

**Figure 4:**
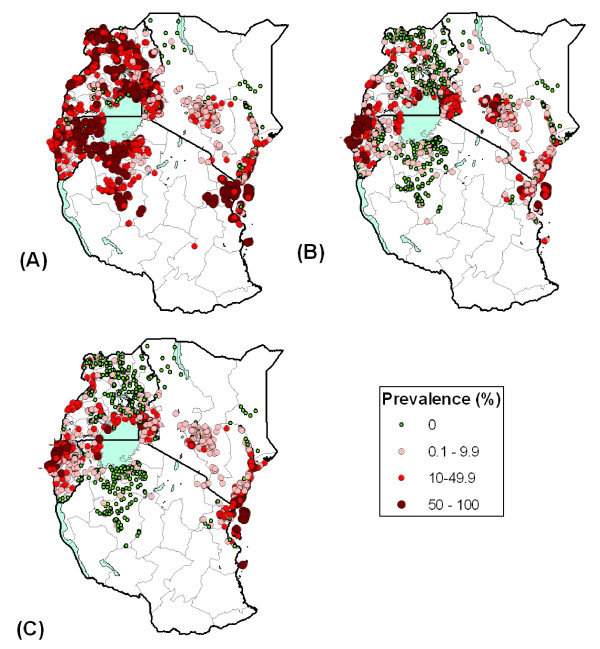
**The known geographical distribution of soil-transmitted helminths in East Africa**. The geographical distribution of (A) hookworm, (B) *Ascaris lumbricoides*, and (C) *Trichuris trichuira*, based on available survey collected between 1980 and 2009, and categorized according to WHO prevalence thresholds (*n *= 1,948). First-level administrative boundaries are indicated in grey. The relatively wide distribution of hookworm is apparent in most surveyed areas in East Africa, except in northern Kenya and northeast Uganda. The distribution of *A. lumbricoides *and *T. trichuria *infection is more restricted, with high prevalence estimates reported in Burundi and Rwanda, central and western Kenya, southeast Uganda, northeast Tanzania and Zanzibar (Pemba and Unguja).

Figures 3 and 4 present the geographical distribution of infection prevalence in East Africa for each of the major helminth species, based on the included survey data. *S. haematobium *was most prevalent along the Kenyan and Tanzanian coast, along Tana River in Kenya, and near Lake Victoria in Kenya and Tanzania (Figure 3A). *S. mansoni *is the only schistosome species in Burundi and Rwanda, and the dominant species in Uganda, where it typically occurs along the shores of large lakes, a pattern also evident in western Kenya and northwest Tanzania (Figure 3B). Of the STH species, hookworm is the most widely distributed species occurring throughout much of East Africa, except in northern Kenya and northeast Uganda (Figure 4A). In contrast, *A. lumbricoides *and *T. trichuria *have much more restricted distributions, though similar to each other, with highest prevalences found in Burundi, central and western Kenya, southeastern Uganda, northeastern Tanzania and Zanzibar (Pemba and Unguja) (Figure 4B and 4C).

Estimates of median prevalence varied considerably between countries and regions within a country (Additional File 1). Data on all species of STH were available for all of the eight provinces in Kenya except Nairobi; 13 (50%) of the 26 provinces in Tanzania; and all of the four regions in Uganda. The highest reported estimates of STH prevalence were respectively found in Western Province in Kenya; North Pemba and South Pemba in Tanzania; and Eastern and Western regions in Uganda. Data on the prevalence of *S. haematobium*, were available for all regions in Kenya except for Central, Nairobi and Western regions, as well as 14 (54%) of the provinces in Tanzania, and three (75%) of the regions in Uganda. The highest median prevalence of *S. haematobium *was at the Coast (33.1%) in Kenya and Mwanza (58.3%) in Tanzania. In Uganda, *S. haematobium *was only found in a small focus north of Lake Kyoga. *S. mansoni *prevalence data were reported from all regions in Kenya, with the exception of Nairobi and North Eastern regions, as well as in nine (35%) of the provinces in Tanzania, and all regions in Uganda. The Eastern region of Kenya had the highest median prevalence for *S. mansoni *(32.1%), while in Tanzania the highest median prevalence was in Unguja North (73.3%) and in Uganda found in the Northern region (17.3%).

## Discussion

These analyses confirm that there is considerable geographical variation in the occurrence of helminth infections in East Africa, and that geographically targeted control programs are required to maximize the cost-effectiveness of chemotherapy. In the absence of easily accessible estimates, the identification of priority areas for control and determination of drug requirements has often been based on unsystematic or out-of-date information, with negative consequences for efficiency and cost-effectiveness. As an extreme example, absence of evidence has sometimes resulted in deworming being included as a component of public health efforts in areas where helminth infection was rare or absent. Building on our previous work [18], we have used newly refined search and geo-positioning strategies to develop an updated atlas of human helminth infection in East Africa. The assembled database represents the largest survey collation for the sub-region, including some 2,612 estimates of infection prevalence, with the majority of surveys conducted since 2000. More than half of these surveys were identified from unpublished sources, confirming the importance of this exercise in providing policy makers and public health planners with access to data. This also indicates that easily searchable biomedical databanks are an insufficient resource, and that an essential step in data collation is an active search in the region and the countries through unpublished reports and theses, as well as following up on personal communications. The collated information serves both to describe the geographical distribution of different helminth species in the sub-region and to identify the relatively very few areas where further data are required.

While considering the value of the mapping approach it is also important to recognize the inherent limitations, which relate to the collection of data, geo-positioning of the points, and differences in survey methodology. Sparseness of data for many regions within Kenya and Tanzania limits the precision of current estimates and highlights the need to identify additional unpublished data or to undertake surveys to fill the gaps in our spatial understanding. Another potential bias in the presented maps arises from the parasitological method employed. Detection of STH or *S. mansoni *infection was mainly based on a single Kato-Katz smear, which may miss light infections because of poor sensitivity and day-to-day fluctuation in egg excretion [44,45], and multiple smears are recommended where possible [46]. Delays in processing samples after collection may also introduce bias, although this aspect is more important for hookworm than for *S. mansoni *[47]. The accepted gold standard of *S. haematobium *detection is urine filtration through a hydrophilic, polycarbonate membrane, but single filtrations may limit sensitivity due to high day-to-day variation in egg counts [48]. Finally, the use of urine reagent strips for diagnosing *S. haematobium *is known to have a lower sensitivity, especially among women of reproductive age due to contamination of urine with vaginal blood (for a review see [49]). While these factors should be borne in mind in interpreting local data, they are unlikely to have a major effect at a policy decision level.

The observed geographical distribution supports historical opinion about infection in rural East Africa and our understanding of the biological determinants of helminth transmission [7,42,50-53]. Specifically, the prevalence of *S. haematobium *is greatest along the coast and along the Tana River in Kenya, whereas *S. mansoni *is notably absent from these areas. Schistosomiasis in Burundi and Rwanda is exclusively due to *S. mansoni *and the same species is dominant in Uganda, accounting for >99% of schistosome infections. In these three countries, *S. mansoni *is most prevalent along the shores of large lakes. Also, there is an absence of transmission of either schistosome species in northern Kenya and in highland areas throughout the region. The absence of *S. mansoni *on the coast and absence of either schistosome species in northern Kenya is probably due to thermal exclusion [54]. It is suggested that geographical differences in compatibility between schistosome parasites and snail intermediate hosts may explain the apparent absence of *S. haematobium *in Uganda [55,56]. Geographical variation in temperature and humidity may also explain, in part, the observed distribution of STH species, with hookworm exhibiting a higher thermal tolerance and hence more widespread geographical distribution [27,57].

It should be noted that the collated data do not accurately reflect transmission patterns among peri-urban and urban populations, where current surveys are particularly lacking. Although there are often differences in infection prevalence between urban and rural communities, they seldom occur in a systematic manner [27], and there are examples where helminth transmission is lower in urban areas than rural areas, for example, in Kampala in Uganda [58,59]. Given the rapid rate of urbanization in East Africa, as elsewhere in the developing world, there is an urgent need for more comprehensive information on helminth infection in urban populations.

Our search strategies identified a few papers which included potentially useful information, but which did not provide sufficient detail on either survey methodology or results to be included into the atlas. Authors of these studies were contacted directly for additional information, where contact addresses could be identified, but emails either did not reach their destination or were not replied. Therefore, to avoid future lost of potentially useful information, we recommend a minimum content which is to be presented in survey reports and publications:

- Number examined

- Number infected

- Date of survey

- Age range and sex of population examined

- Method of stool and urine examination

- Name of school or community

- Longitude and latitude of school or community

- District and region in which survey was undertaken

The information contained in the atlas also highlights areas for which no suitable data were identified, including all much of southern and western Tanzania. In order to help collect suitable prevalence data, there are a number of scientific approaches which can be employed to rapidly and effectively map the distribution of helminth infection (see [49] for a review). A challenge for current survey approaches is how to best define a nationally representative sampling strategy, which takes into account (i) population density, (ii) known ecological correlates of infection which can help exclude areas where transmission is unlikely to occur, and (iii) the existence of previous data. This is a non-trivial issue, and requires careful statistical consideration. Moreover, sampling should not necessarily be defined by administrative boundaries. It may be pragmatic to define sampling in relation to the distribution of schools or health facilities which can deliver interventions; this approach can help create ownership and allow for a more efficient implementation of control. The increasingly availability of spatial national health facility databases [60] and school databases [61] should inform such sampling considerations.

The usefulness of the data presented here could be further enhanced by the production of risk maps created using Bayesian model-based geostatistics [62], and enabling prediction of the prevalence of infection with each schistosome and STH species even in as yet unsurveyed areas, and potentially across the continent. Such maps have been created at the sub-national [63-65], national and regional scales [66-68]. However, there has been no Bayesian geostatistical risk maps of helminths reported at the continental scale, such as the map recently reported for malaria [16]. While we have presented ecological regression-based maps for the African continent [27], model-based geostatistical risk maps will provide a more flexible tool for estimating spatial heterogeneity in disease risk and disease burden, together with associated uncertainties. Information on uncertainty is useful for prioritizing future data collection and assessing risks associated with different resource allocation strategies [68]. Overlays of continental risk maps for various important diseases would potentially enable assessment of the need for and potential impact of integrated control programmes.

## Conclusion

For East Africa, the information assembled in the current atlas provides the most reliable, up-to-date and comprehensive source of data on the distribution of common helminth infections. Such information is invaluable to help guide the rational implementation of control efforts and estimation of drug requirements. As countries continue to implement control, we will collaborate with national and international implementing partners to provide updated iterations of the maps. We are also working to develop similar maps for the whole of sub-Saharan Africa and for other helminth endemic areas of the world. A final goal is to make the information easily accessible in the public domain based on an open access information platform. This is the subject of ongoing efforts.

## Abbreviations

Abbreviations are used in the text, tables or figures: (DVBD): Division of Vector Borne Diseases; (GIS): geographic information systems; (GRUMP): Global Rural Urban Mapping Project; (MSHs): Medical Subject Headings; (MoH): Ministry of Health; (SALB): Second Administrative Boundaries; (STH): soil-transmitted helminth; (SSA): sub-Saharan Africa; (WHO): World Health Organization.

## Competing interests

The authors declare that they have no competing interests.

## Authors' contributions

SB and DAPB conceived the experiments, with later input from ACAC and RWS. SB, JLS, DM and PK compiled, mapped and analysed the prevalence data. NBK, MM, DM, ON, NJSL, CM and EM contributed to data collation and provided a control programme perspective. SB wrote the first draft of the manuscript. All other authors commented on the final draft of the manuscript. All authors have read and approved the final manuscript.

## Supplementary Material

Additional file 1 **Estimates of median species-specific prevalence by countries and by regions within a country**. The median, inter-quartile range (IQR), minimum and maximum estimates of infection prevalence by helminth species and by region for Kenya, Tanzania and Uganda, 1980–2009. Click here for file
