# Supplementary material for: An updated atlas of human helminth infections: the example of East Africa
Source: Int J Health Geogr. 2009 Jul 9;8:42. doi: 10.1186/1476-072X-8-42 (PMC2714505; doi:10.1186/1476-072X-8-42)
Supplement: Additional file 1 — Estimates of median species-specific prevalence by countries and by regions within a country. The median, inter-quartile range (IQR), minimum and maximum estimates of infection prevalence by helminth species and by region for Kenya, Tanzania and Uganda, 1980–2009. [file 1476-072X-8-42-S1.doc]

## Additional Information.

## The table below presents the median, inter-quartile range (IQR), minimum and maximum estimates of infection prevalence by helminth species and by region for Kenya, Tanzania and Uganda.

## Table. The median prevalence of species infection (inter-quartile range, minimum and maximum) (*n*) by country for Burundi and Rwanda and by first-level administrative boundary for Kenya, Tanzania and Uganda, 1980-2009.

|  | Hookworm | *A. lumbricoides* | *T. trichuira* | *S. haematobium* | *S. mansoni* |
| --- | --- | --- | --- | --- | --- |
|  |  |  |  |  |  |
| **Burundi** | **20.0 (11-28, 2, 52) (31)** | **8.3 (3-17, 0, 62) (31)** | **6.7 (2-19, 0, 52) (31)** | **0.0 (31)** | **5.0 (0-13, 0, 58) (41)** |
|  |  |  |  |  |  |
| **Rwanda** | **30.2 (12-48, 0, 88) (134)** | **28.3 (3-72, 0, 97) (134)** | **9.9 (3-49, 0, 97) (134)** | **0.0 (134)** | **0.0 (0-2, 0, 70) (138)** |
|  |  |  |  |  |  |
| **Kenya** | **12.5 (4-33, 0, 100) (847)** | **14.3 (3-36, 0, 91) (842)** | **6.9 (2-20, 0, 98) (785)** | **23.3 (6-50, 0, 98) (625)** | **8.0 (1-25, 0, 100) (661)** |
| Nairobi | - | - | - | - | - |
| Central | 5.1 (1-13, 0, 44) (176) | 15.0 (1-46, 0, 75) (176) | 0.9 (0-2, 0, 20) (161) | - | 10.5 (1-46, 0, 94) (128) |
| Coast | 15.1 (1-50, 0, 100) (173) | 9.1 (2-29, 0, 69) (173) | 15.7 (6-40, 0, 98) (163) | 33.5 (13-59, 0, 98) (391) | 0.0 (0-0, 0, 62) (79) |
| Eastern | 5.3 (3-11, 0, 41) (58) | 3.2 (1-9, 0, 27) (50) | 1.8 (1-3, 0, 32) (47) | 6.8 (3-20, 0, 67) (24) | 32.1 (6-64,0, 98) (73) |
| North Eastern | 0.0 (0-0, 0, 0) (17) | 0 (0-0, 0, 2) (17) | 0.0 (0-0, 0, 1) (15) | 3.0 (0-11, 0, 44) (15) | - |
| Nyanza | 17.6 (8-33, 0, 91) (349) | 18.5 (7-37, 0 , 91) (352) | 11.9 (4-20, 0, 71) (329) | 10.4 (0-30, 0, 92) (194) | 8.5 (2-19, 0, 98) (312) |
| Rift Valley | 1.3 (0-7, 0, 30) (23) | 0.9 (0-12, 0, 43) (21) | 0.3 (0-2, 0, 13) (20) | 0.0 (0-0, 0, 0) (1) | 0 (0-5, 0, 22) (25) |
| Western | 79.4 (65-85, 31, 95) (51) | 32.0 (25-55, 0, 89) (53) | 40.2 (29-70, 9, 96) (50) | - | 15.3 (7-38, 1, 100) (44) |
|  |  |  |  |  |  |
| **Tanzania** | **49.6 (30-73, 0, 100) (321)** | **0 (0-11, 0, 94) (319)** | **0.0 (0-13, 0, 100) (319)** | **31.1 (7-55, 0, 96) (345)** | **1.6 (0-5, 0, 95) (267)** |
| Arusha | - | - | - | - | - |
| Dar-es-Salaam | 19.6 (7-32, 7, 32) (2) | 4.0 (1) | 0.0 (1) | 16.2 (13-43, 5, 55) (13) | - |
| Dodoma | - | - | - | - | - |
| Iringa | - | - | - | - | - |
| Kagera | 71.7 (60-78, 27, 93) (33) | 3.3 (0-10, 0, 53) (33) | 1.7 (0-13, 0, 55) (33) | 0.0 (0-8, 0, 67) (33) | 1.7 (0-7, 0, 78) (33) |
| Kigoma | - | - | - | - | - |
| Kilimanjaro | 3.5 (3-9, 2, 14) (4) | 7.2 (4-10, 1, 13) (4) | 6.7 (3-13, 0, 18) (4) | 35.1 (1-44, 0, 53) (6) | 29.4 (23-51, 1, 73) (5) |
| Lindi | - | - | - | - | - |
| Mara | - | - | - | - | - |
| Mbeya | - | - | - | - | - |
| Morogoro | 21.0 (1) | - | - | 10.7 (5-24, 0, 67) (33) | - |
| Mtwara | - | - | - | 25.8 (1) | - |
| Mwanza | 40.2 (30-53, 7, 95) (78) | 0 (0-0, 0, 7) (78) | 0.0 (0-0, 0, 3) (78) | 58.3 (43-72, 5, 92) (79) | 5.0 (2-17, 0, 95) (83) |
| Pwani | 59.3 (43-74, 35, 88) (9) | 5.6 (2-7, 0, 15) (9) | 5.7 (0-11, 0, 61) (9) | 52.0 (39-62, 29, 65) (7) | 0.0 (0-0, 0, 0) (9) |
| Rukwa | - | - | - | - | - |
| Ruvuma | - | - | - | - | - |
| Shinyanga | 20.0 (5-47, 0, 90) (35) | 0 (0-0, 0, 3) (35) | 0.0 (0-0, 0, 0) (35) | 36.1 (25-57, 2, 90) (35) | 0.0 (0-2, 0, 7) (35) |
| Singida | - | - | - | - | - |
| Tabora | 43.3 (37-53, 2, 73) (51) | 0 (0-0, 0, 3) (51) | 0.0 (0-0, 0, 3) (51) | 5.0 (2-12, 0, 43) (51) | 0.0 (0-2, 0, 3) (51) |
| Tanga | 67.7 (44-80, 16, 100)(52) | 10.1 (4-21, 0, 61) (52) | 4.9 (1-14, 0, 61) (52) | 48.0 (31-67, 11, 96) (69) | 0.0 (0-0, 0, 52) (49) |
| North Pemba | 95.1 (86-97, 71, 99) (11) | 50.0 (31-62, 5, 82) (11) | 91.4 (78-95, 68,100) (11) | 1.6 (1) | 1.6 (1) |
| South Pemba | 81.2 (52-93, 11, 97) (30) | 56.4 (43-67, 19, 94) (30) | 90.1 (79-96, 66, 99) (30) | - | - |
| Unguja North | 9.1 (0-16, 0, 22) (8) | 25.0 (12-37, 8, 50) (8) | 35.1 (27-56, 19, 73) (8) | 5.3 (0-54, 0, 83) (8) | 73.3 (1) |
| Unguja South | 9.1 (7-12, 7, 12) (2) | 5.7 (2-9, 2, 9) (2) | 41.7 (39-44, 39, 44) (2) | 1.0 (0-4, 0, 15) (8) | - |
| Unguja Urban W | 4.8 (5-13, 0, 16) (5) | 0 (0-0, 0, 6) (5) | 19.6 (5-20, 0, 29) (5) | 42.5 (1) | - |
| Mjini-Magharibi | - | - | - | - | - |
|  |  |  |  |  |  |
| **Uganda** | **25.8 (3-55, 0, 100) (571)** | **0.0 (0-3, 0, 96) (572)** | **0.0 (0-5, 0, 86) (571)** | **0.0 (0-0, 0, 10) (66)** | **6.5 (0-36, 0, 100) (656)** |
| Central | 6.5 (0-30, 0, 86) (146) | 0 (0-3, 0, 50) (147) | 1.7 (0-9, 0, 56) (146) | 0.0 (0-0, 0, 0) (19) | 3.2 (0-11, 0, 100) (168) |
| Eastern | 43.7 (18-70, 0, 100) (188) | 0 (0-0, 0, 42) (188) | 0.0 (0-2, 0, 40) (188) | 0.0 (0-0, 0, 0) (9) | 4.8 (2-42, 0, 100) (205) |
| Northern | 32.2 (2-55, 0, 87) (151) | 0 (0-0, 0, 38) (151) | 0.0 (0-0, 0, 22) (151) | 0.0 (0-0, 0, 10) (35) | 17.3 (5-40, 0, 95) (178) |
| Western | 25.0 (10-44, 0, 90) (86) | 14.4 (0-36, 0, 96) (86) | 11.3 (3-34, 0, 86) (86) | 0.0 (0-0, 0, 0) (3) | 3.0 (0-62, 0, 97) (105) |
